# Analysis of the Influence of Production Method, Plastic Content on the Basic Performance of Waste Plastic Modified Asphalt

**DOI:** 10.3390/polym14204350

**Published:** 2022-10-15

**Authors:** Haibin Li, Lichang Zhou, Jianmei Sun, Sirui Wang, Mingming Zhang, Yihong Hu, Ahmed Abdulakeem Temitope

**Affiliations:** 1 School of Architecture and Civil Engineering, Xi’an University of Science and Technology, Xi’an 710054, China; 2 Shaanxi Transportation Holding Group Co., Ltd., Xi’an 710064, China; 3 School of Material Science and Engineering, Chang’an University, Xi’an 710064, China

**Keywords:** road engineering, waste plastic, polypropylene (PP), polyethylene (PE), modified asphalt, basic performance, mechanism analysis, SBS modified asphalt

## Abstract

The sustainable reuse of waste plastic as an alternative construction material has numerous environmental and economic advantages. New opportunities to recycle waste plastic in asphalt for road construction would mitigate landfill issues and significantly reduce global carbon emissions. With a clear aim to contribute to a more efficient reuse of waste plastic, this paper reutilized two types of waste plastic (polypropylene (PP) and polyethylene (PE)) as asphalt modifiers to improve the performance of asphalt pavement as well as to achieve the purpose of sustainable recycling waste plastic. Therefore, the optimal preparation parameters of plastic-modified asphalt were recommended by the orthogonal test. Then, the dispersion and modification mechanisms of plastic particles in plastic-modified asphalt were further studied by Fourier Transform Infrared Spectroscopy (FTIR) and Thermogravimetric Differential Scanning Calorimetry (TG-DSC). The results show that the asphalt containing PP and PE shows better overall performance at high temperatures compared with the base asphalt. Furthermore, PE-modified asphalt and PP-modified asphalt exhibited optimal properties when prepared at 3000 rpm for 30 min at 170 °C. Moreover, the results of the expansion mechanism show that the main reaction process of plastic asphalt is a physical change. Finally, PP-modified asphalt and PE-modified asphalt generally perform well and are suitable for high-temperature areas. Consequentially, the results of this research promote the recycling of waste plastic, ultimately advocating the recycling of waste materials and environmental protection of pavement construction.

## 1. Introduction

Asphalt pavement is widely used for its smooth surface and driving comfort. However, with the high temperature in southern and western China in summer, as well as the sudden increase in traffic volume, heavy overload, and drainage traffic, asphalt pavement defects are becoming more and more prominent [1]. The properties of base asphalt are not so good that the road asphalt maintenance budget every year exceeds the budgetary allocation for new asphalt pavement construction. It has become clear that these base asphalt pavements are incapable of meeting the high-temperature stability, low-temperature crack resistance, anti-aging, and durability requirements of modern transportation. Therefore, it is necessary to choose a more effective, more economic, and simpler asphalt modifier to improve its performance [2].

The production and usage of plastic products have made great contributions to almost all different industries as well as exacerbating the waste of resources and increasing pollution worldwide. The management of waste plastic remains a global challenge for developed and developing countries alike. Plastic pollution, also known as “white pollution”, causes crop yield reduction, animal deaths, and land occupation, which affect sustainable land use. China, the world’s largest consumer of plastics, is facing a huge challenge now. More than 63 million tons of plastic had been produced in this country by 1.4 billion people in 2021 alone; the recycling rate is only about 30%, which results in the filling of landfills at an alarming rate [3]. Efficient and economical approaches to dealing with these waste plastics have been urgently needed. Currently, attention to the field of waste recycling has continuously increased. Waste plastic is one of the most popular types. The successful application of recycled plastic would significantly reduce plastic pollution, improve efficiency in the utilization of resources, and decrease the carbon footprint. Additionally, it is an effective way to reduce production costs.

As high molecular polymers, plastics are generally divided into thermosetting plastic and thermoplastic. The physical and chemical properties of plastic are stable and can be used for the modification of base asphalt. Due to the stable physical and chemical properties of plastic, the usage of waste plastic, such as polyethylene (PE) [4,5,6], high-density polyethylene (HDPE) [7], low-density polyethylene (LDPE) [7], polypropylene (PP) [8,9,10], ethylene-vinyl acetate (EVA) [11], polyvinyl chloride (PVC) [12], polyethylene terephthalate (PET) [13,14], and polystyrene (PS) to improve the performance of asphalt is one of the research hotspots in road engineering. Many factors including construction ability, availability, cost, and expected performance are important in the choice of modifier for asphalt. Therefore, from an environmental and economic point of view, the use of recycled waste plastic materials instead of raw materials could have several advantages such as help easing landfill pressures and reducing demands of extraction from quarries and other natural sources.

The application of recycled waste plastic-modified asphalt, whether it is EVA, PP, or PE-modified asphalt, will effectively change the structure of raw asphalt collage and form new collage structures, thus improving the high-temperature resistance, moisture susceptibility, and other properties of the asphalt mixture, and furthermore improving the pavement quality, saving maintenance costs, and extending the service life of asphalt pavement.

Modarres and Hamedi [13] applied shredded PET fine particles to asphalt mixes and the results showed that PET particles improved the fatigue behavior and stiffness of the asphalt mixes. Xu et al. [14] found that the overall properties of rubber asphalt could be improved by the application of chemically recycling waste PET. Jianmin Ma et al. [7] analyzed the effects of four typical recycled plastics (including HDPE, LDPE, PP, and PS) on the performance of asphalt, it was found that high-temperature binder grades improved while low-temperature grades were relatively unaffected. Plastics increase strength but reduce strain tolerance in ductile failure. So, the study proposed that only binder course layers should be modified to prevent undue risk for premature and excessive surface cracking. Dalhat and Al-Abdul Wahhab [9] reported that following extensive research, PP and PE significantly improved the rutting and fatigue performance as well as the resilient modulus (MR) of base asphalt. Hınıslıoğlu and Ağar [15] concluded that for optimum Marshall stability, flow, and Marshall quotient (MQ), 4% PE should be mixed with base asphalt at 165 °C for 30 min. PE existed in many variations; Ho, et al. [16] found that PE with lower molecular weight and wider molecular weight distribution were more suitable materials for asphalt modification. Through exhaustive research, Costa et al. [17,18] confirmed the superiority of EVA and PE-modified asphalt over base asphalt. Köfteci [19] proved that 4% PE material content gave modified asphalt better stability values than base asphalt. Martin-Alfonso, et al. [20] indicated that PE considerably improved the viscoelastic properties as well as water sensitivity and fatigue resistance of ordinary base asphalt. Abed and Bahia [21] also noted that PE obviously improved the penetration grade as well as the strength properties of base asphalt, when compared with an unmodified asphalt mixture. Khurshid, et al. [22] found that PE-modified asphalt had better stability, rutting resistance, and load-bearing capacity when used in asphalt mixtures. In other research, Mansourian, et al. [23] reported better low-temperature properties and rutting resistance in asphalt following EVA and PE dosing.

Although there are many studies on using waste plastic as asphalt modifiers, few of them consider the climatic factors of the used areas. Problems caused by high temperatures in combination with high traffic volume and heavy loads remain to be solved. On the other hand, improper disposal of waste plastic causes harm to water bodies and soil, and therefore, rationally, it must be used in order to realize its sustainable development. Thus, the objective of this paper is to comprehensively study the reutilization of waste plastic (PP and PE) in asphalt and to improve the performance of asphalt in high-temperature areas. The optimal preparation parameters of plastic-modified asphalt are recommended by orthogonal test. Then, the dispersion and modification mechanism of plastic in modified asphalt is further studied by Fourier Transform Infrared Spectroscopy (FTIR) and Thermogravimetric Differential Scanning Calorimetry (TG-DSC). The evaluation of plastic-modified asphalt will enable further enhancement of the performance of the pavement. This would also bring new knowledge to the construction materials industry to develop new products for different environments and accelerate the reuse of waste resources.

## 2. Materials and Methods

### 2.1. Materials

#### 2.1.1. Asphalt

This paper selected the South Korean 90# (SK-90#) base asphalt to prepare the modified asphalts. The basic performance indexes of the SK-90# asphalt were shown in Table 1.

#### 2.1.2. Plastic

**Figure 1:**
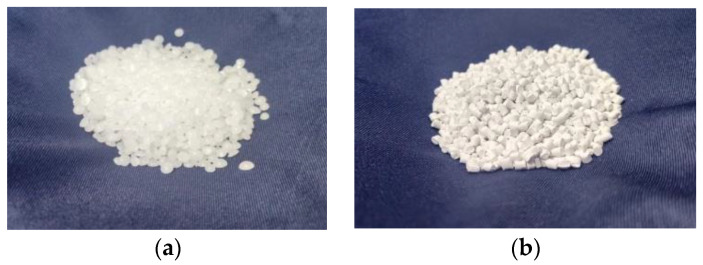
Appearances of recycled PE pellets and PP pellets. (**a**) PE pellets (**b**) PP pellets.

In this paper, two different kinds of waste plastic (PE and PP) were selected which had a large consumption at present. The corresponding physical appearances were shown in Figure 1, and the physical and chemical indexes were shown in Table 2 and Table 3, respectively.

#### 2.1.3. Preparation of Plastic Modified Asphalt

**Figure 2:**
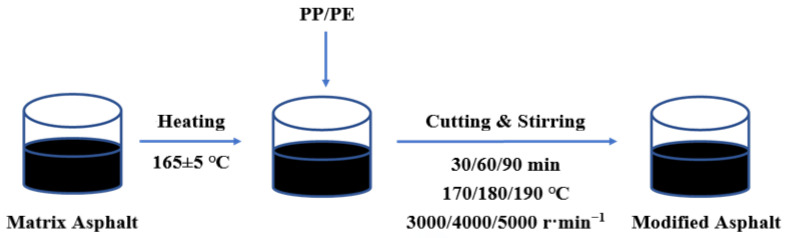
Preparation of plastic-modified asphalt.

According to the preparation process and relevant technical specifications of modified asphalt, the plastic content, shearing time, shearing temperature, and shearing speed were selected as key parameters in the preparation process of plastic-modified asphalt [24]. The specific preparation process is as follows:

As is shown in Figure 2, the base asphalt was heated to 165 °C ± 5 °C until it turned into liquid. Then, according to the research [25,26], the appropriate amount of PE (0, 2%, 4%, 6%, 8%) and PP (0, 2%, 4%, 6%, 8%, 10%, 12%) was predetermined to add into the base asphalt. Nine groups of specimens with no less than three specimens in each group were prepared under different shearing times (30 min, 60 min, 90 min), shearing temperatures (170 °C, 180 °C, 190 °C), and shearing rates (3000 r/min, 4000 r/min, 5000 r/min).

In this study, SBS-modified asphalt was simultaneously prepared for comparative analysis of plastic-modified asphalt. The mixing time was 45 min, and the temperature was limited to 175 ± 5 °C. A shearing rate of 3000 r/min was adopted. SBS content was maintained at 5% by weight, the standard value was based on Standard Test Methods of Bitumen and Bituminous Mixtures for Highway Engineering (JTG E20-2011).

### 2.2. Experimental Methods

#### 2.2.1. Orthogonal Test

To optimize the composition of plastic-modified asphalt, an orthogonal test was adopted in this study. Penetration, softening point, and viscosity were selected as the main indicators to measure the basic physical properties of plastic-modified asphalt. The parameters (shearing time, shearing temperature, and shearing rate) were taken as control factors that affected the properties of plastic-modified asphalt. After the preliminary test of the research team, different shearing times (30 min, 60 min, 90 min), shearing temperatures (170 °C, 180 °C, 190 °C), and shearing rates (3000 r/min, 4000 r/min, 5000 r/min) in the preparation process of plastic modified asphalt were taken as cross-testing to explore the law and influence of various factors of plastic modified asphalt. The orthogonal test scheme is shown in Table 4.

#### 2.2.2. Physical Property Tests

According to the test methods T0604-2011, T0606-2011, T0625-2011, and T0605-2011 in the JTG E20-2011, the penetration, softening point, viscosity, and ductility tests of plastic-modified asphalt were carried out.

1. Penetration refers to the vertical penetration distance of a standard needle under constant load (100 g) for 5 s at a temperature of 25 °C. It is measured in 0.1 mm units and is used to assess the hardness of asphalt.
2. The softening point is the average value of the temperature. The steel ball passes through the asphalt plate and falls from a height. When the steel ball just touches the bottom plate at a specified distance, the water temperature at this time is the softening point of the asphalt.
3. The 135 °C viscosity is a basic characteristic that reflects the frictional resistance between the internal molecules of asphalt during flow deformation.
4. The 5 °C ductility refers to the length at which the prepared sample is stretched at a certain temperature and a certain speed to break.

#### 2.2.3. Fourier Transform Infrared Spectroscopy (FTIR)

FTIR belongs to the molecular absorption spectrum, which is widely used in the qualitative and quantitative analysis of organic materials [27]. In order to verify the effect of plastic from the microscopic point of view, the functional groups of the plastic-modified asphalt and base asphalt were observed and tested by an infrared spectrometer. The FTIR (Nicolet Is10 Fourier Transform Infrared Spectrometer produced by Thermo Scientific, Madison, WI, United States) was used to determine the different functional groups of plastic and plastic-modified asphalt in wave numbers ranging from 4000 cm^−1^ to 500 cm^−1^. The samples were cut into small pieces and placed in the IR cell.

#### 2.2.4. Thermogravimetric Differential Scanning Calorimetry (TG-DSC)

The thermal stability of plastic-modified asphalt was analyzed by thermogravimetric analysis (TG) and differential scanning calorimetry (DSC). There are mainly two technologies used in this test, differential scanning calorimetry, and thermogravimetric analysis, which can detect the physical changes and chemical reactions of the samples in the experimental process. The comprehensive heat analytical instrument was used to analyze the base asphalt and the plastic-modified asphalt. The heating rate and nitrogen flow rate of the test were set to 10 °C/min and 20 mL/min, respectively, and the temperature was continuously increased to 800 °C.

## 3. Results and Discussion

### 3.1. Orthogonal Test Analysis

#### 3.1.1. Optimal Preparation Parameters of PE Modified Asphalt

According to the orthogonal test in Table 4, a total of nine groups of orthogonal tests were carried out, and the results were shown in Table 5. The variance analysis results of the orthogonal test were listed in Table 6, Table 7 and Table 8. In order to better analyze the orthogonal test results, the sum of factor test results *K* and extreme difference *R* is proposed to describe the data. *K_i_* is the sum of values, and column-level number is *i*. In addition, the variance of the three levels is *R* (extreme difference) which is used to quantify the influencing degree of factors to the test index. The larger of *R* value, the greater impact of the factor on the test parameter.

Table 6, Table 7 and Table 8 listed the effect of different preparation parameters on the penetration, softening point, and viscosity of PE-modified asphalt. From Table 7 and Table 8, according to the *R* values, the factors that affect the penetration and viscosity were shearing rate, shearing time, and shearing temperature, in that order. However, the temperature became the foremost factor affecting the softening point, as the *R*-value of the shearing temperature (4.5) exceeds that of the shearing rate (1.0). Based on Table 6, Table 7 and Table 8, it can be explained that the shearing rate, shearing time, and shearing temperature had some negative effects on the properties of specimens. Table 6 displayed the softening point with various factors. The large values of *K* represented the high softening point that indicated less sensitivity to the high-temperature properties of plastic-modified asphalt. According to the *K* values in Table 6, the softening point was the best when the shearing time, shearing temperature, and shearing rate were 30 min, 170 °C, and 3000 rpm, respectively. From the above analysis, the optimal preparation parameters of PE-modified asphalt were determined, in which the shearing time was 30 min, the shearing temperature was 170 °C, and the shearing rate was 3000 rpm.

#### 3.1.2. Optimal Preparation Parameters of PP Modified Asphalt

According to the orthogonal test scheme in Table 4, a total of 9 groups of orthogonal tests are carried out, and the test data results were shown in Table 9.

The variance analysis results of the orthogonal tests were listed in Table 10, Table 11 and Table 12. Table 10, Table 11 and Table 12 listed the effect of different preparation parameters on the penetration, softening point, and viscosity of PP-modified asphalt. From Table 10 and Table 12, according to the *R* values, the factors affecting the softening point and viscosity were shearing temperature, shearing time, and shearing rate, in that order. However, the shearing rate became the foremost factor affecting penetration, as the *R*-value of the shearing rate (4.1) exceeds that of temperature (4.0). Based on the data in Table 10, Table 11 and Table 12, it can be explained that the shearing rate, shearing temperature, and shearing time also had some negative effects on the properties of specimens. Table 10 displayed the softening point of specimens with various factors. The large values of *K* represented the high softening point that indicated less sensitivity to the high-temperature properties of plastic-modified asphalt. According to the *K* values in Table 10, when the shearing time, shearing temperature, and shearing rate were 30 min, 170 °C, and 3000 rpm, respectively, the softening point was the best. From the analysis above, the optimal preparation parameters of PP-modified asphalt, and the optimal preparation parameters of the specimen were determined: the shearing time was 30 min, the shearing temperature was 170 °C, and the shearing rate was 3000 rpm.

### 3.2. Influence of Plastic Contents on Physical Properties of Base Asphalt

#### 3.2.1. Effect of PE Contents on the Modified Asphalt

**Figure 3:**
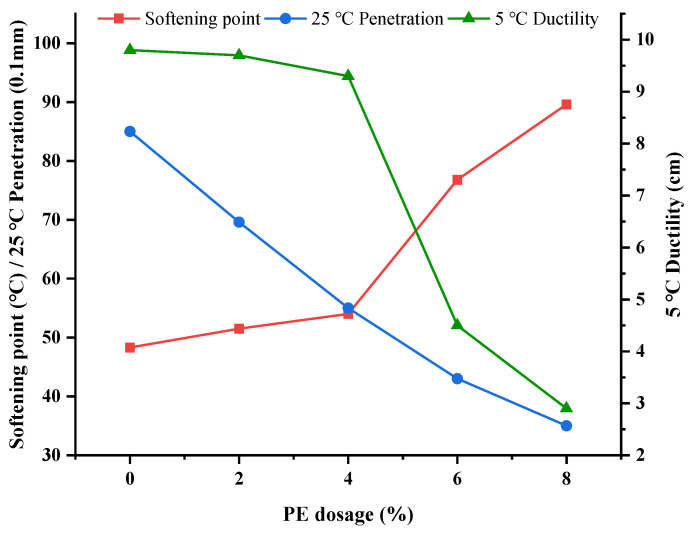
Influence curve of softening point, penetration, and ductility with different PE dosages.

According to the previous studies [28,29,30], different dosages (0, 2%, 4%, 6%, 8%) of PE were selected, and PE-modified asphalt was prepared with the optimal preparation parameters. Figure 3 shows the curves of softening point, penetration, and ductility of PE- modified asphalt with various contents of PE.

It can be seen from Figure 3 that the softening point was improved after adding PE. When the content of PE increased from 4% to 6%, the softening point showed a significant increase of 42%. The reason was that when PE content was increased, phase separation appeared, showing that the plastic phase was distributed in the continuous phase of asphalt. In this way, the plastic phase was swelled by absorbing the light components in asphalt, thereby improving the performance of modified asphalt. It can be seen that the penetration decreased after adding PE. The reason was that the combination of plastics in asphalt formed a good three-dimensional network structure, which also made asphalt harden, finally leading to a decrease in penetration. Meanwhile, the ductility of asphalt was decreased. When the content of PE increased from 4% to 6%, the ductility at 5 °C had an obvious decrease in a leap of 52%. With the increase of the plastic contents, some defects on the surface or inside would produce tiny holes under the action of stress concentration and then develop into very fine lines. When the deformation developed further and the straight orientation molecular chain was broken, it would turn into micro cracks. The small particles of plastic played the role of stress concentration, and the modifier particles caused a large number of fine lines or shear bands under external tension, thus reducing the room temperature crack resistance of PE-modified asphalt.

#### 3.2.2. Effect of PP Contents on the Modified Asphalt

**Figure 4:**
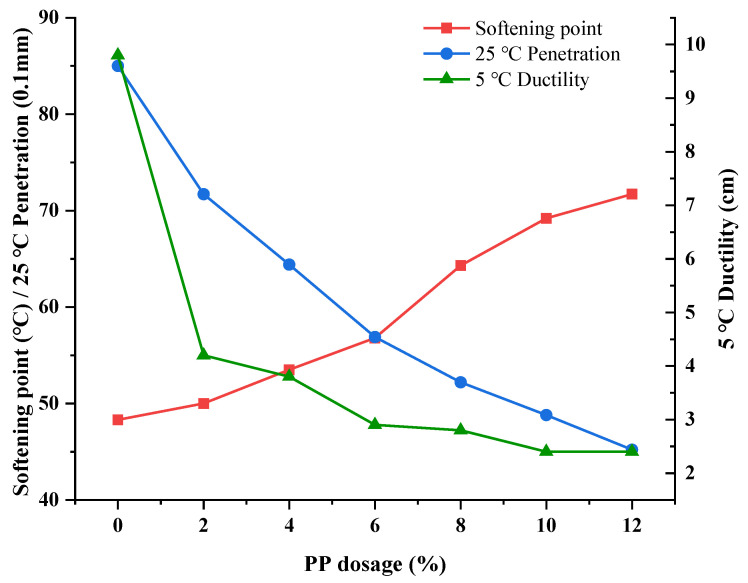
Influence curve of softening point, penetration, and ductility with different PP dosages.

According to the previous study [31], different dosages (0, 2%, 4%, 6%, 8%, 10%, 12%) of PP were selected, and the PP-modified asphalt was prepared with the optimal preparation parameters. The curves of softening point, penetration, and ductility are shown in Figure 4.

It can be seen from Figure 4 that the softening point of asphalt was obviously improved after adding PP. The softening point decreased as the dosage of PP increased. When the PP content was less than 6%, the softening point increased slowly, and when the PP content increases from 6% to 8%, the softening point of the plastic-modified asphalt increases significantly, and the increment is about 14%, and when the PP content increases from 8% to 10%, the softening point rises slowly. It can be seen that the penetration and 5 °C ductility of PP-modified asphalt decrease with the increase in PP dosage. At low content, plastic improved the penetration of base asphalt, however, with the increasing of plastic, the content of oxidized substances (such as resins or asphaltenes) began to accumulate, which damaged the structure of the asphalt components and inhibited the stretching effect of the performance of base asphalt.

The softening point of asphalt increased and the penetration decreased for the PE and PP-modified asphalts with the contents increasing. However, when the content of PE and PP was less than 8%, the low-temperature ductility decreased slightly after the initial decreasing trend. In addition, when the PP content was higher than 8%, the ductility decreased by 0.4, while the softening point increased by 4.9 and penetration decreased by 3.7. Furthermore, in order to maximize the improvement effects of PE and PP on asphalt performance in high-temperature areas, the recommended optimal dosages of PE and PP were 5% and 9% respectively.

### 3.3. Comparative Analysis of Basic Performances with SBS Modified Asphalt

**Figure 5:**
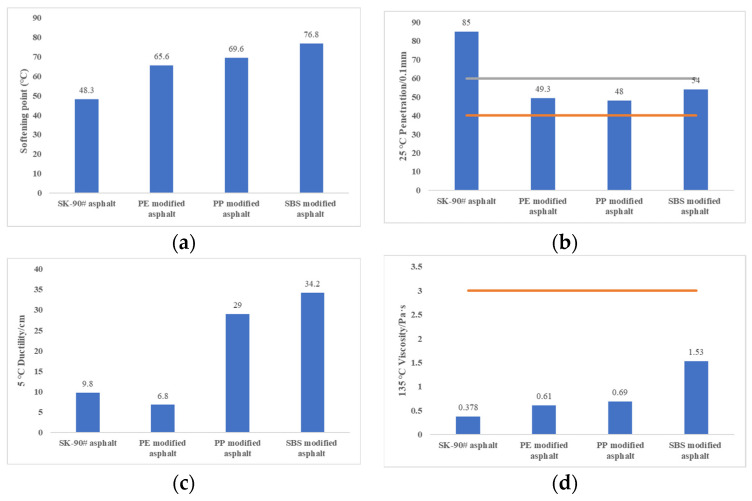
Comparative analysis of matrix asphalt and SBS modified asphalt. (**a**) Softening point. (**b**) Penetration. (**c**) Ductility. (**d**) Viscosity.

With the high-temperature melt mixing method, PE-modified asphalt, and PP-modified asphalt were prepared under the optimal preparation parameters and optimal dosage, respectively. Compared with base asphalt and SBS-modified asphalt, the results of penetration, softening point, ductility, and viscosity were shown in Figure 5.

Figure 5 shows that all the indexes adequately meet the specification requirements. Moreover, with the addition of PE and PP, the softening point increased by 35.8% and 44.1% respectively, which indicated better high-temperature properties of plastic-modified asphalt. However, the SBS modifier had a more significant influence on base asphalt, increasing the softening point of asphalt by 59.1%. The penetration decreased by 42% and 43.5% respectively, which has similar effects with the SBS modifier on the asphalt. Meanwhile, the highest influence of these modifiers could be on the viscosity of the asphalt [32]. PE-modified asphalt and PP-modified asphalt increased the viscosity of asphalt by 61.4% and 82.5% respectively, indicating good workability and consistency in road construction. Furthermore, the softening points of the PE-modified asphalt and PP-modified asphalt are obviously higher than that of base asphalt, while the penetration was lower than that of base asphalt, which indicates the better high-temperature performance of plastic-modified asphalt. When compared with the SBS modifiers, the waste plastic modifiers adequately meet the requirements of the specification, whilst only lagging behind these expensive, specially produced modifiers, by small margins. This further confirms the efficacy of plastic modifiers as viable economical substitutes for their expensive counterparts.

### 3.4. Mechanism Analysis of Plastic-Modified Asphalt

#### 3.4.1. Effects of Functional Groups on Plastic-Modified Asphalt

The functional groups of asphalt can be divided into the following characteristic peaks: the strong absorption at 2880~2980 cm^−1^ is the C-H stretching vibration absorption peak of cycloalkanes and alkanes, the variable angle vibration absorption peaks of CH_3_ and CH_2_ is at 1450 cm^−1^ and 1370 cm^−1^, the strong stretching vibration absorption band of aromatic C=C and multi conjugated hydrogen bound C=O are at about 1600 cm^−1^. The presence of Carboxylic acid, Ketone, and Quinone is shown at the 1700 cm^−1^ absorption peak, and the absorption peak at 700–900 cm^−1^ shows the out-of-plane vibration of aromatic components.

**Figure 6:**
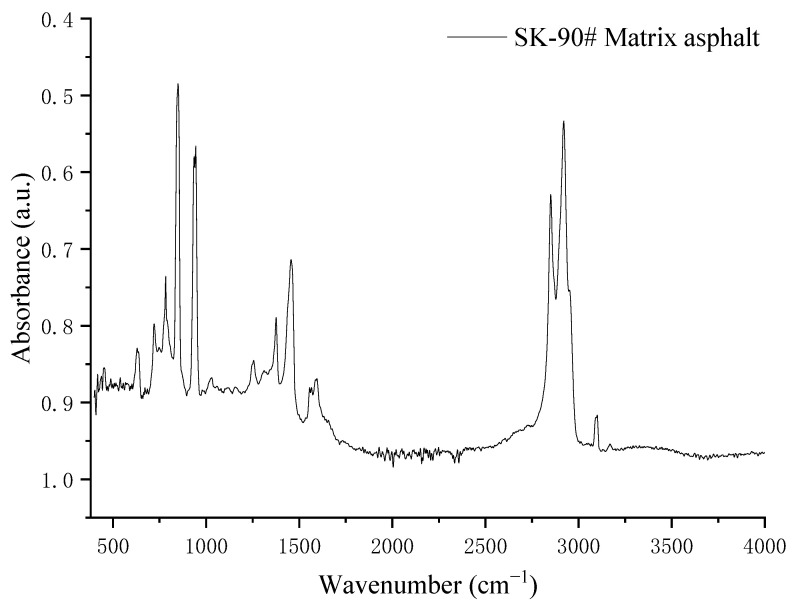
FTIR image of SK-90# matrix asphalt.

As shown in Figure 6, the SK-90# base asphalt was characterized by strong absorption peaks of C-H, stretching vibration from 2850 cm^−1^ to 2990 cm^−1^; an obvious absorption peak of CH_2_ variable angle vibration stretching was from 760 cm^−1^ to 960 cm^−1^ and CH_3_ variable angle vibration was near 1480 cm^−1^.

**Figure 7:**
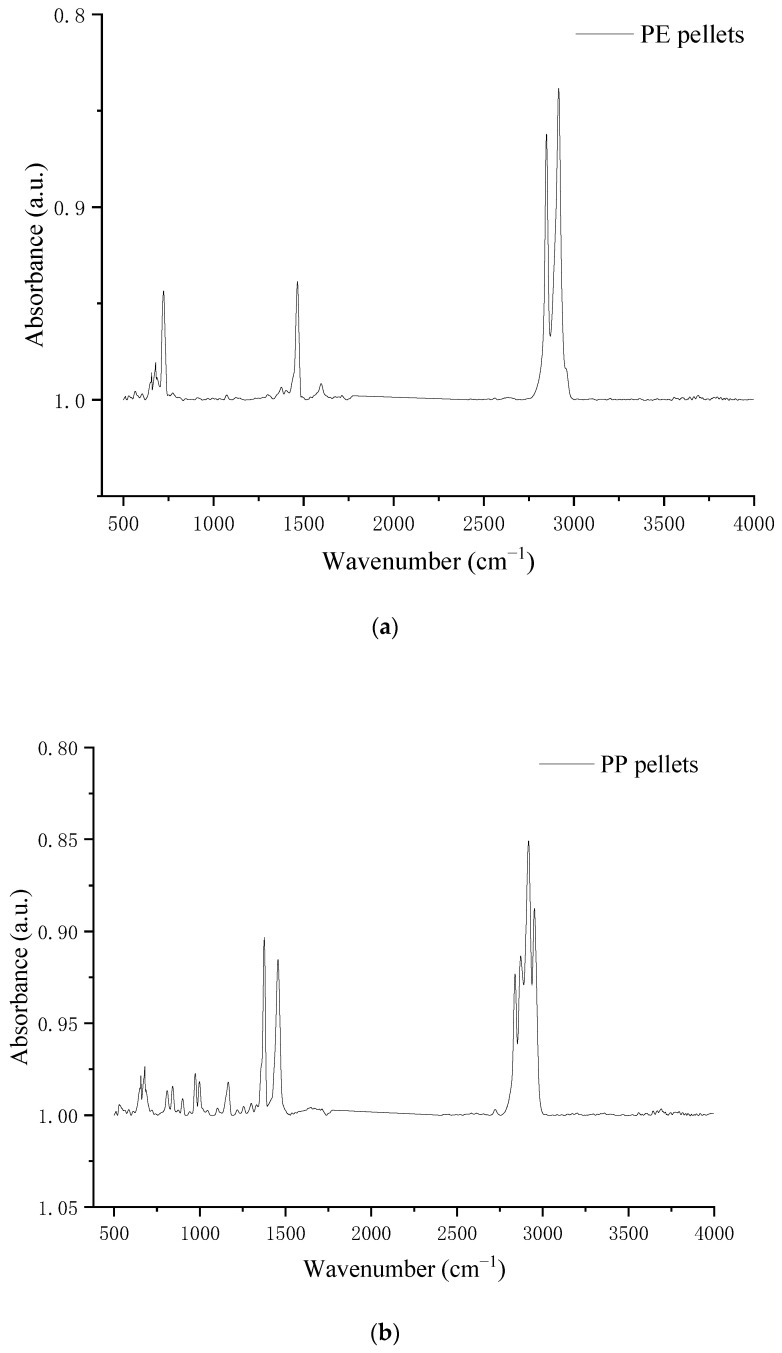
The functional groups change of different plastic pellets. (**a**) PE pellets. (**b**) PP pellets.

The main characteristic peaks of PE and PP were depicted in Figure 7. The strong absorption peaks from 2850 cm^−1^ to 2990 cm^−1^ were C-H stretches vibration of PE, and the absorption peaks of CH_3_ and CH_2_ variable angle vibration were near 1480 cm^−1^ and 760 cm^−1^. Meanwhile, PP also showed strong absorption peaks of C-H stretching vibration from 2850 cm^−1^ to 2990 cm^−1^, but it was characterized by the absorption peaks of CH_3_ and CH_2_ variable angle vibration stretching from 1280 cm^−1^ and 1490 cm^−1^ which was unlike the PE.

**Figure 8:**
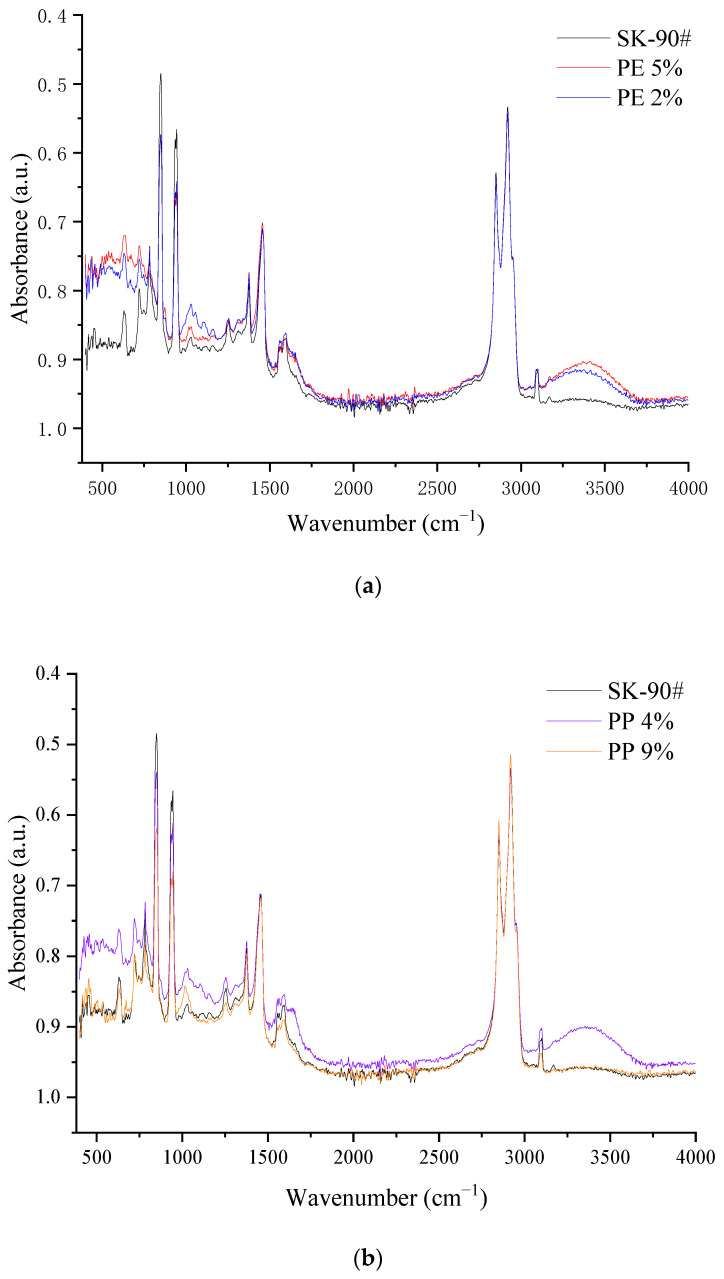
The functional groups change of plastic-modified asphalt and matrix asphalt. (**a**) PE-modified asphalt and SK-90#. (**b**) PP-modified asphalt and SK-90#.

The main characteristic peaks of plastic-modified asphalt and base asphalt were depicted in Figure 8. When the asphalt was modified with PE and PP, the out-of-plane vibration of aromatic components was obvious, such as the C-H stretching vibration absorption peaks of cycloalkanes and alkanes at 2850 cm^−1^ to 2990 cm^−1^, the CH_3_ and CH_2_ variable angle vibration absorption peaks at 1450 cm^−1^ and 1370 cm^−1^, and the absorption peaks at 700~900 cm^−1^. New absorption peaks were not found in the whole functional group region when compared with base asphalt. Meanwhile, in Figure 8b, there was a stronger absorption band in the range of 2760~3000 cm^−1^ which was very close to the position of the infrared absorption peak of the base asphalt. Moreover, some absorption peaks even coincide with the base asphalt which showed there was no chemical reaction between plastic and base asphalt. The mixing process was a mainly physical reaction. The PE and PP absorbed light components in asphalt and swelled during the shearing process.

#### 3.4.2. Thermogravimetric Analysis

**Figure 9:**
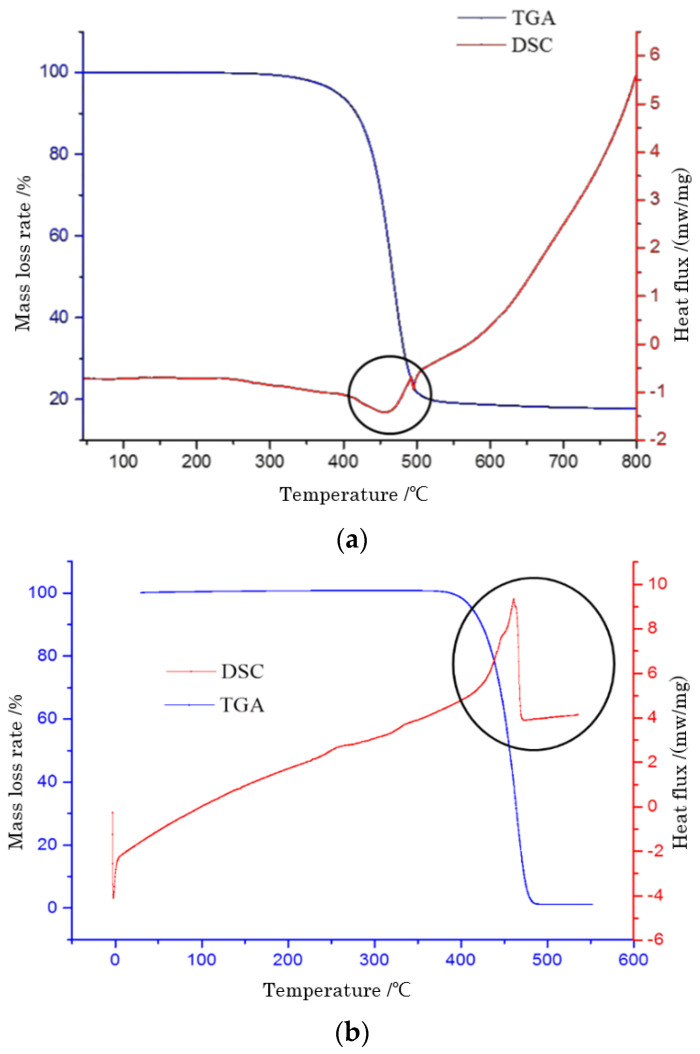
TG-DSC spectrum of base asphalt and PE-modified asphalt. (**a**) TG-DSC spectrum of base asphalt. (**b**) TG-DSC spectrum of PE modified asphalt.

**Figure 10:**
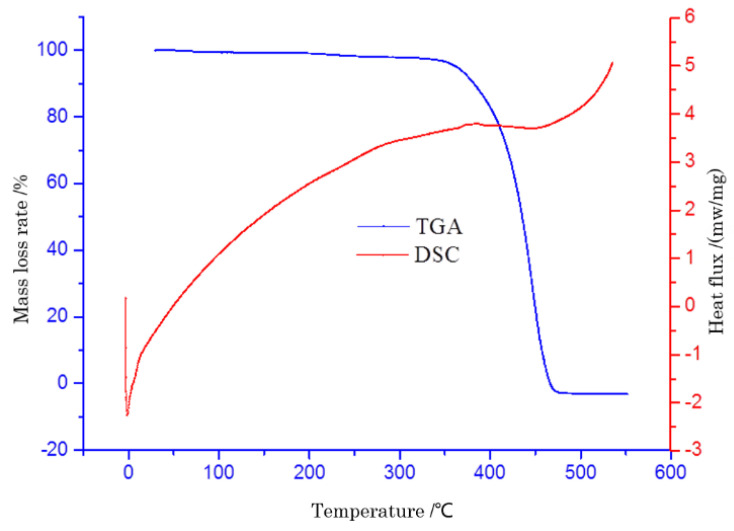
TG-DSC spectrum of PP-modified asphalt.

**Figure 11:**
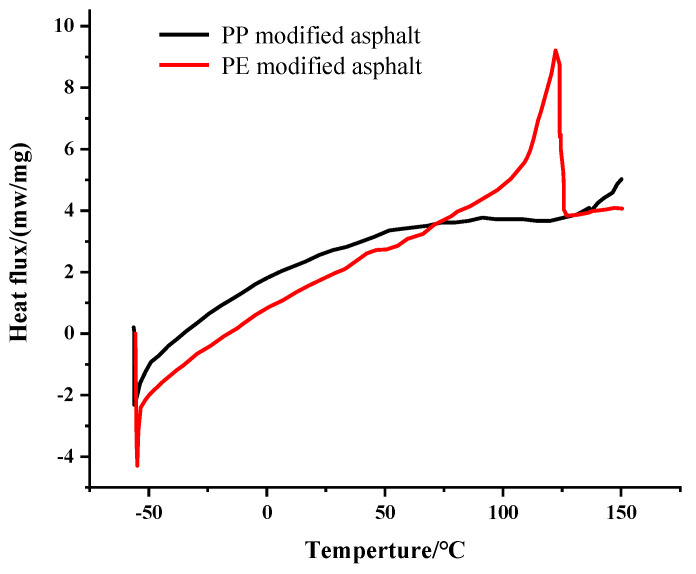
Thermal analysis curve of plastic-modified asphalt.

Figure 9, Figure 10 and Figure 11 were the TG-DSC curves of base asphalt, PE-modified asphalt, and PP- modified asphalt, respectively. Figure 9a showed the TG-DSC spectrum of SK-90# base asphalt. The TG curve presented a downward trend which was three weight loss stages in the test process of base asphalt. The first weight loss stage occurred in the temperature range of 150~400 °C, and the weight loss was about 10%.

After 290 °C, the weight of base asphalt decreased obviously, which meant that base asphalt underwent a process of combustion during the mass loss. The second stage of mass loss occurred at 380~540 °C. In this stage, base asphalt lost 80% of its weight, which was also the most mass loss among the three stages. When the temperature reached 560 °C, base asphalt tended to be stable and the residual mass of asphalt was 17.6%. From the curve, the base asphalt was endothermic and exothermic at 0~40 °C and 400~600 °C, respectively. It can be found that the exothermic peak appeared at 460 °C with the maximum mass loss rate of base asphalt. In the process of rapid mass change, a large number of chemical reactions and the exothermic reactions occurred in the complex components of base asphalt. The concentration time of the exothermic process was short due to the complex chemical reaction.

Figure 9b showed the thermal analysis spectrum of PE-modified asphalt. It can be seen that the thermal analysis spectrum of PE-modified asphalt was obviously different from the base asphalt. Firstly, from the TG curve, it can be seen that the mass of the test sample showed a downward trend. When the temperature rose to about 360 °C, the mass loss of asphalt began to change obviously. At this time, the test sample began to decompose by heating, and the curve changed gently until the test temperature was about 500 °C. This was because some physical changes occurred in the PE pellets beyond 360 °C. Some components with similar structures in the asphalt were adsorbed by these PE particles. Under the action of some lightweight components, PE pellets swelled, and the structural composition of the asphalt was changed, which increased its viscosity and changed the polyethylene state from a crystalline state to an amorphous state. Compared with the temperature range of 290 °C to 500 °C, the PE-modified asphalt mass loss was diminished by 33.3% from 360 °C to 500 °C, which indicated that the process of PE-modified asphalt mass loss was shorter. It showed that the addition of PE pellets reduced the contents of light components, such as saturated phenanthrene and aromatic components. Additionally, it increased the contents of asphaltene and gum in base asphalt under high-temperature melting, which made the asphalt have better high-temperature performance.

Figure 10 shows the trend of the thermal analysis spectrum of PP-modified asphalt which was similar to that of PE-modified asphalt. However, the changing trend of PP-modified asphalt began earlier. The main reason was that the lightweight components in the base asphalt were absorbed by the PP pellets. Due to the high-speed shearing action, the PP pellets were scattered in the base asphalt. During this process, the required energy was less in the range of room temperature to 600 °C, which indicated that the PP pellets started the physical reaction much easier than PE pellets. Although the DSC curve changed very little, there was still some mass loss from inception until 360 °C, which was still similar to PE-modified asphalt. When the test temperature rose to about 460 °C, the mass loss of the test sample tended to be flat. All the results were consistent with the previous research conclusions.

As shown in Figure 11, PE-modified asphalt had a larger endothermic peak area than PP-modified asphalt. When the plastic pellets were added, the DSC curve of the corresponding modified asphalts became smoother, and the corresponding peak was smaller, which could prove that the temperature stability of PE-modified asphalt was more stable than that of PP-modified asphalt. The reason was that the addition of PE pellets made the asphalt components more complex, the swelling and cross-linking reaction between plastic and asphalt was more sufficient under high-temperature conditions, the contents of light components in the asphalt were reduced, and the PE pellet was not completely swollen, existing as small particles in the modified asphalt.

Meanwhile, with the temperature increasing, the adsorption between molecules made the remaining components in the asphalt have no obvious change, and the DSC curve only showed a gentle peak. When the temperature was between −40 °C and 30 °C, the asphalt changed from a solid state to a liquid state with performance changing. At this time, the performance of asphalt mainly depended on the solid components part. In this temperature range, the exothermic peak of PE-modified asphalt was naturally larger than that of PP-modified asphalts because its temperature sensitivity was obvious.

Furthermore, when the modified asphalt changed from a solid state to a liquid state, the volume of flowing components increased and the intermolecular force decreased rapidly. Due to the existence of pellets, the light components in plastic-modified asphalt existed in crystallized forms. When the temperature increased, the amount of solid matter and components in this part of the asphalt changed obviously. That meant the phase state with an obvious exothermic peak changed sharply. As a result, these plastic-modified asphalts were more suitable to be used in high-temperature regions.

## 4. Conclusions

Introducing waste plastics into the asphalt as a modifier was one of the most innovative, reasonable, and effective ways to recycle them owing to the economic benefits and reduced negative impact on the environment. Therefore, this paper put forward a way of using waste PE and PP as asphalt modifiers. The optimal process conditions and preparation parameters were proposed, the performance of PE-modified asphalt and PP-modified asphalt were studied and the reaction mechanism was revealed. The conclusions were summarized as follows:

(1) The performance of base asphalt can obviously be affected by PE and PP. The proper preparation parameters were 30 min shearing time, 170 °C shearing temperature, and 3000 r/min shearing rate. The optimal dosage for the PE and PP were recommended to be 5% and 9% respectively.
(2) When compared with SBS modifiers, the waste plastic, adequately met the requirements of the relevant specification, whilst only lagging behind these expensive specially produced modifiers, by small margins. As in the case of the softening point, where the waste plastic modified asphalts were only lower by an average of 8.2% than the special modifiers, indicating the efficiency of waste plastic modifiers as an economical substitute.
(3) The plastic-modified asphalt and the base asphalt had similar functional groups, especially the positions of the C-H stretching absorption peaks, CH_3_, and CH_2_ variable angle absorption peaks of the cycloalkanes and alkanes. There were no new absorption peaks which indicated that the mixing process of asphalt and waste plastic was a physical swelling reaction.
(4) The plastic-modified asphalt showed better thermal stability with a mass loss of about 33.3%. Due to the absorbing and light-reducing components of asphalt, the mass loss process of plastic-modified asphalt was shorter. The temperature sensitivity of waste plastic-modified asphalt was reduced since the endothermic peak area and exothermic peak area of the DSC curve increased obviously.
(5) The good high-temperature performance of plastic-modified asphalt made it more suitable in high-temperature regions. Since PE was the most produced plastic type, it is recommended to reuse it as a modifier in asphalt; it will therefore achieve an additional environmental conservation aim and provide a new way for the recycling of waste materials.

The preparation, modification mechanism, and basic properties of PE and PP-modified asphalt were focused on in this study, and further studies will focus on more types of plastic, low-temperature performance improvement, and compatibility. In particular, the use of molecular simulation techniques is strongly recommended for further investigation of asphalt compatibility and modification mechanisms at the molecular level.

## Figures and Tables

**Table 1:** Basic performance indexes of SK-90# asphalt.

Test		Unit	Measured Value	Specification Requirement
Penetration (25 °C, 100 g, 5 s)		0.1 mm	85	80~100
Ductility (5 cm/min, 15 °C)		cm	>100	>100
Softening point		°C	48.3	>45
Density (15 °C)		g/cm^3^	1.030	
Aging (TFOT) test (163 °C, 5 h)	Quality change	%	0.120	≤±0.8
	Residual penetration	%	73.6	≥57
	Residual ductility	cm	11.7	≥8

**Table 2:** Basic indicators of recycled PE pellets.

Density/g·cm^−3^	Melting Point/°C	Melt Rate/g·10 min^−1^	Elongation Break Rate/%
0.91~0.92	43	0.2~5.0	20~800

**Table 3:** Basic indicators of recycled PP pellets.

Density/g·cm^−3^	Melting Point/°C	Elongation Break Rate/%
0.90~0.91	135~159	150~600

**Table 4:** Orthogonal test design table.

Tests	Shearing Time/min	Shearing Temperature/°C	Shearing Rate/r·min^−1^
l	30	170	3000
2	30	180	4000
3	30	190	5000
4	60	170	4000
5	60	180	5000
6	60	190	3000
7	90	170	5000
8	90	180	3000
9	90	190	4000

**Table 5:** Properties of PE-modified asphalt.

Factor	Softening Point/ °C	25 °C Penetration/0.1 mm	135 °C Viscosity/Pa·s
Test l	65.1	47.0	0.618
Test 2	61.9	47.3	0.609
Test 3	63.6	46.4	0.628
Test 4	63.3	47.5	0.606
Test 5	62.9	47.3	0.612
Test 6	62.6	48.4	0.600
Test 7	62.5	47.2	0.617
Test 8	61.6	49.3	0.594
Test 9	63.1	47.4	0.609

**Table 6:** PE Calculation results of softening point test *R* and *K* values of PE modified asphalt.

Factor	Shear Time/min	Shear Temperature/°C	Shear Rate/r·min^−1^	Softening Point/°C
Test l	30	170	3000	65.1
Test 2	30	180	4000	61.9
Test 3	30	190	5000	63.5
Test 4	60	170	4000	63.3
Test 5	60	180	5000	62.9
Test 6	60	190	3000	62.6
Test 7	90	170	5000	62.5
Test 8	90	180	3000	61.6
Test 9	90	190	4000	63.1
*K*_1_	190.5	190.9	189.3	/
*K*_2_	188.8	186.4	188.3	/
*K*_3_	187.2	189.2	188.9	/
*R*	3.3	4.5	1.0	/

**Table 7:** Calculation results of penetration test *R* and *K* values of PE modified asphalt.

Factor	Shear Time/min	Shear Temperature/°C	Shear Rate/r·min^−1^	25 °C Penetration/0.1 mm
Test l	30	170	3000	47.0
Test 2	30	180	4000	47.3
Test 3	30	190	5000	46.4
Test 4	60	170	4000	47.5
Test 5	60	180	5000	47.3
Test 6	60	190	3000	48.4
Test 7	90	170	5000	47.2
Test 8	90	180	3000	49.3
Test 9	90	190	4000	47.4
*K*_1_	140.1	141.7	144.7	/
*K*_2_	143.2	143.9	142.2	/
*K*_3_	143.9	142.2	140.9	/
*R*	3.8	2.2	3.9	/

**Table 8:** Calculation results of viscosity test *R* and *K* values of PE modified asphalt.

Factor	Shear Time/min	Shear Temperature/°C	Shear Rate/r·min^−1^	135 °C Viscosity/Pa·s
Test l	30	170	3000	0.618
Test 2	30	180	4000	0.609
Test 3	30	190	5000	0.628
Test 4	60	170	4000	0.606
Test 5	60	180	5000	0.612
Test 6	60	190	3000	0.600
Test 7	90	170	5000	0.617
Test 8	90	180	3000	0.594
Test 9	90	190	4000	0.609
*K*_1_	1.855	1.814	1.812	/
*K*_2_	1.818	1.815	1.823	/
*K*_3_	1.820	1.827	1.857	/
*R*	0.037	0.012	0.045	/

**Table 9:** Properties of PP-modified asphalt.

Factor	Softening Point/°C	25 °C Penetration/0.1 mm	135 °C Viscosity/Pa·s
Test l	63.4	54.5	0.670
Test 2	61.6	52.3	0.679
Test 3	60.9	51.8	0.682
Test 4	62.6	51.6	0.684
Test 5	61.4	51.4	0.687
Test 6	60.8	51.3	0.687
Test 7	62.0	52.5	0.676
Test 8	61.2	51.0	0.690
Test 9	60.6	51.5	0.685

**Table 10:** PE Calculation results of softening point test *R* and *K* values of PP modified asphalt.

Factor	Shear Time/min	Shear Temperature/°C	Shear Rate/r·min^−1^	Softening Point/°C
Test l	30	170	3000	63.4
Test 2	30	180	4000	61.6
Test 3	30	190	5000	60.9
Test 4	60	170	4000	62.6
Test 5	60	180	5000	61.4
Test 6	60	190	3000	60.8
Test 7	90	170	5000	62.0
Test 8	90	180	3000	61.2
Test 9	90	190	4000	60.6
*K*_1_	185.9	188.0	185.4	/
*K*_2_	184.8	184.2	184.8	/
*K*_3_	183.8	182.3	184.3	/
*R*	0.7	1.9	0.37	/

**Table 11:** Calculation results of penetration test *R* and *K* values of PP modified asphalt.

Factor	Shear Time/min	Shear Temperature/°C	Shear Rate/r·min^−1^	25 °C Penetration/0.1 mm
Test l	30	170	3000	54.5
Test 2	30	180	4000	52.3
Test 3	30	190	5000	51.8
Test 4	60	170	4000	51.6
Test 5	60	180	5000	51.4
Test 6	60	190	3000	51.3
Test 7	90	170	5000	52.5
Test 8	90	180	3000	51.0
Test 9	90	190	4000	51.5
*K*_1_	158.4	158.6	156.8	/
*K*_2_	154.3	154.7	155.4	/
*K*_3_	155.0	154.6	155.7	/
*R*	4.1	4.0	1.4	/

**Table 12:** Calculation results of viscosity test *R* and *K* values of PP-modified asphalt.

Factor	Shear Time/min	Shear Temperature/°C	Shear Rate/r·min^−1^	135 °C Viscosity/Pa·s
Test l	30	170	3000	0.670
Test 2	30	180	4000	0.679
Test 3	30	190	5000	0.682
Test 4	60	170	4000	0.684
Test 5	60	180	5000	0.687
Test 6	60	190	3000	0.687
Test 7	90	170	5000	0.676
Test 8	90	180	3000	0.690
Test 9	90	190	4000	0.685
*K*_1_	2.031	2.030	2.047	/
*K*_2_	2.028	2.056	2.048	/
*K*_3_	2.051	2.054	2.045	/
*R*	0.023	0.026	0.003	/

## Data Availability

All the data used in this article are in the manuscript.
